# A Phase III study (CheckMate 238) of adjuvant immunotherapy with nivolumab (NIVO) versus ipilimumab (IPI) after complete resection of stage IIIb/c or stage IV melanoma (MEL) in patients (pts) at high risk for recurrence

**DOI:** 10.1186/2051-1426-3-S2-P166

**Published:** 2015-11-04

**Authors:** Jeffrey Weber, Jean-Jaques Grob, Kim A Margolin, Paolo A Ascierto, Mario Sznol, Patrick A Ott, Chantal Lejeune, Veerle de Pril, Mary M Ruisi, F Stephen Hodi

**Affiliations:** 1 H. Lee Moffitt Cancer Center, Tampa, FL, USA; 2 Aix-Marseille University, Hopital de la Timone, Marseille, France; 3 Stanford University Medical Center, Valley Village, CA, USA; 4 Istituto Nazionale Tumori Fondazione G. Pascale, Naples, Italy; 5 Yale Comprehensive Cancer Center, New Haven, CT, USA; 6 Dana-Farber Cancer Institute, Boston, MA, USA; 7 Bristol-Myers Squibb, Braine l'Alleud, Belgium; 8 Bristol-Myers Squibb, Princeton, NJ, USA

## Background

There is currently no consensus on the standard of care for pts with completely resected stage IIIB/C or stage IV MEL with no evidence of disease (NED) who are at high risk for recurrence. NIVO, a PD-1 immune checkpoint inhibitor, is approved in the United States, Europe, and Japan as monotherapy for the treatment of pts with advanced MEL and has shown a benefit in overall survival (OS) versus dacarbazine in treatment-naive pts with wild-type *BRAF*, metastatic MEL in a Phase III study. In a Phase I study, NIVO plus a multipeptide vaccine followed by NIVO monotherapy demonstrated promising median recurrence-free survival (RFS; 47.1 months) and median OS (not reached) as adjuvant therapy for high-risk, resected stage IIIC/IV MEL. IPI, a CTLA-4 immune checkpoint inhibitor, is approved as monotherapy for the treatment of pts with advanced MEL and has been shown to improve OS in advanced MEL and increase RFS versus placebo when used at a higher dose and more prolonged schedule in adjuvant therapy of stage III MEL. This Phase III, randomized, placebo-controlled, double-blind study will compare NIVO with IPI as adjuvant therapy in pts with stage IIIB/C and stage IV NED MEL who are at high risk for recurrence.

## Methods

Eligible pts are ≥15 years of age, have stage IIIB/C or IV histologically confirmed MEL that is completely resected, and have been rendered disease free within 12 weeks prior to randomization. Pts having uveal MEL, a history of autoimmune disease, a condition requiring systemic corticosteroids or other immunosuppressive medications, or receiving prior systemic therapy for MEL will be excluded. Pts will be randomized 1:1 to NIVO 3 mg/kg every 2 weeks (Q2W) or IPI 10 mg/kg Q3W for 4 doses then Q12W starting at week 24, and treated until disease recurrence, unacceptable toxicity, or consent withdrawal, for up to 1 year. The primary and secondary objectives are to evaluate RFS and OS, respectively. An estimated 800 pts will be randomized. Clinical Trial registration number: NCT02388906

## Trial registration

ClinicalTrials.gov identifier NCT02388906.

